# Anti-inflammatory effects of an autologous gold-based serum therapy in osteoarthritis patients

**DOI:** 10.1038/s41598-022-07187-3

**Published:** 2022-03-03

**Authors:** Jessica Feldt, Anna-Jasmina Donaubauer, Jessica Welss, Ulrich Schneider, Udo S. Gaipl, Friedrich Paulsen

**Affiliations:** 1 Institute of Functional and Clinical Anatomy, Friedrich Alexander University Erlangen-Nürnberg, Universitätsstr. 19, 91054 Erlangen, Germany; 2 Translational Radiobiology, Department of Radiation Oncology, Universitätsklinikum Erlangen, Friedrich Alexander University Erlangen-Nürnberg (FAU), Universitätsstr. 27, 91054 Erlangen, Germany; 3 iREG, Gmund Am Tegernsee, Germany; 4 Department of Operative Surgery and Topographic Anatomy, Sechenov University, Moscow, Russia

**Keywords:** Autoimmunity, Cytokines, Innate immune cells

## Abstract

Osteoarthritis (OA) involves activation and recruitment of immune cells to affected joints, including the production of pro-inflammatory cytokines. Here, a gold-based autologous serum therapy is investigated for its effect on peripheral blood cell composition and cytokine levels in OA patients. From six OA patients serum and blood samples were collected before and after second therapy treatment for analysis of peripheral blood cell composition as well as cytokine levels compared to control samples. This therapy significantly downregulates CD4^+^ T cells and B cells in OA patients after second treatment compared to healthy controls. Monocytes are significantly upregulated in patients after second treatment Serum IL-9 and TNF-α levels are downregulated in patients after second treatment compared to healthy control serum. The activation status of immune cells was modulated after therapy in patients. Anti-inflammatory effects of the peripheral blood cell composition in OA patients can be seen after therapy treatment. After two treatments IL-9 and TNF-α are significantly downregulated in patient serum. Here, primary data of a new autologous therapy for OA treatment and its modulatory effects on cytokines are presented.

## Introduction

Several immune cell types are known to be involved in the pathology of osteoarthritis (OA). Therefore the analysis of cells out of the peripheral immune system is useful for defining biomarkers and discovering the immunological processes which are driving the development of OA. To date, the detailed pathology of OA is not fully understood and treatment is challenging. New approaches of finding appropriate therapies for OA, such as autologous and platelet rich serum therapies are considered^1^. Autologous conditioned serum therapies (ACS) based on incubation with pre-coated glass beads are already established for OA^2^. They are pain reducing and able to restore quality of live shortly after treatment^3^. Because these therapies circumvent common obstacles, such as systemic toxicity or severe immune reactions and also increase bioavailability, they are a promising approach for OA treatment^1^.

A new therapeutic approach based on ACS incubates autologous blood from patients with gold-microparticles (AuMPs)^4^. Gold is commonly used in OA treatment, as in acupuncture^5^ or implants^6^. It has an antioxidant effect in the synovia and also suppresses joint swelling and cartilage degeneration in animal models^7^. An initial phase 2a open label study in patients with moderate to severe OA has stated the beneficial effect of this therapy^8^. In the present preliminary study, the approach is to elucidate and better understand how this new gold-based autologous serum therapy affects the composition of blood cells of OA patients and how inflammation-related cytokines in the serum are modulated due to treatment.

## Results

**Figure 1:**
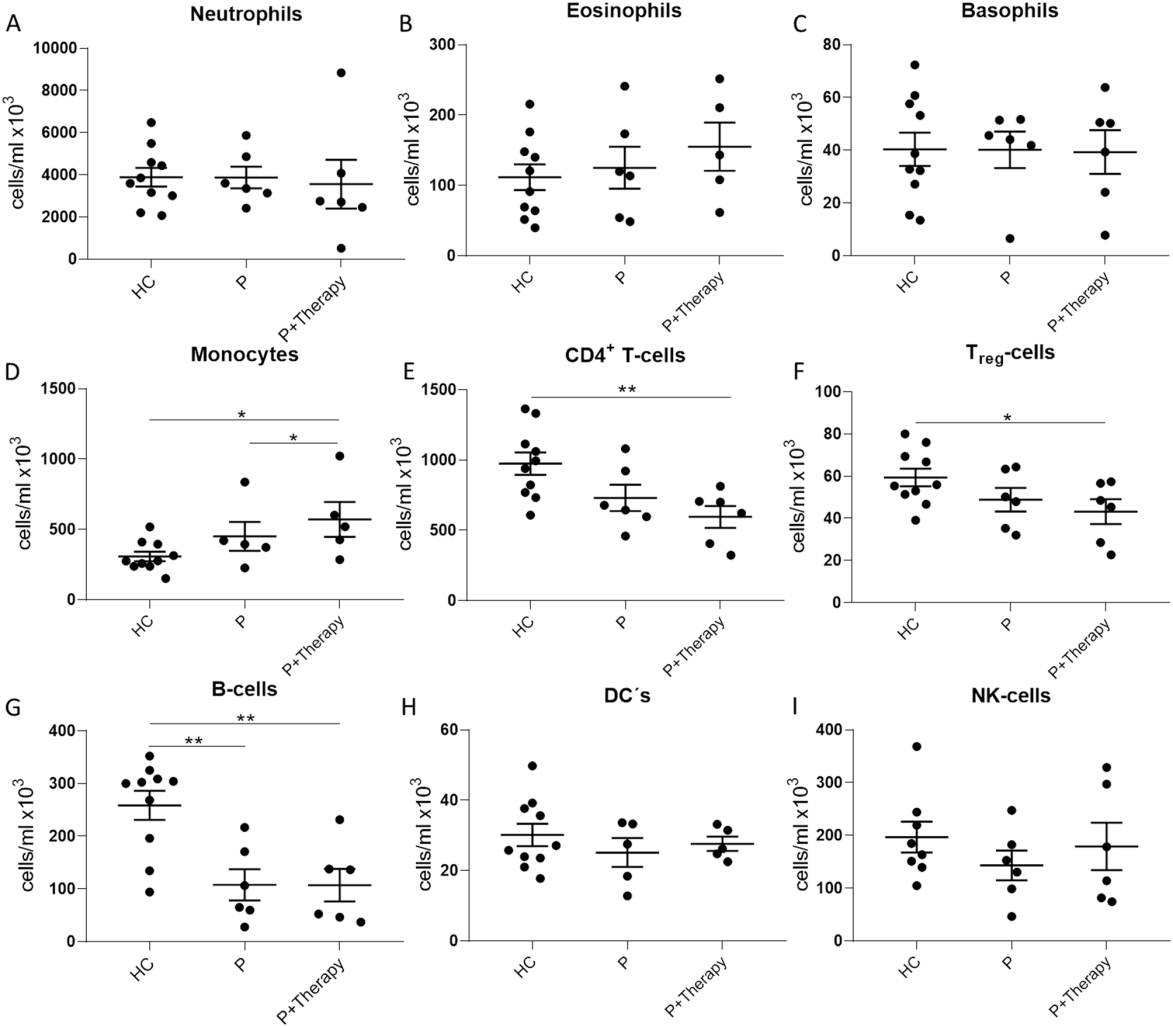
Immunophenotyping analysis of peripheral blood cells from healthy controls and patients. Whole blood samples from healthy controls without any treatment (HC), from OA patients before treatment (P), and from the same patients after the second therapy treatment (P + Therapy) were analysed. The composition of (**A**) neutrophils, (**B**) eosinophils, (**C**) basophils, (**H**) dendritic cells, and (**I**) natural killer cells showed no modulation because of therapy treatment and also no significant differences between healthy controls and patients. CD4^+^ T-cells (**E**) and T_reg_-cells (**F**) revealed a significant decrease in cells after second therapy treatment compared with healthy controls but not with OA patients before treatment. (**G**) B cells were significantly decreased in OA patients in general and (**D**) monocytes showed a significant increase after treatment in OA patients also compared to healthy controls. Results are given as mean ± SEM; T-test, *p < 0.5; **p < 0.01.

To analyse the effect of gold-based autologous serum therapy on the peripheral blood cell composition, we analysed healthy controls (Ctrl), the blood of OA patients before therapy treatment (OA), and the blood of the same patients after the second treatment by immunophenotyping. Figure 1 shows that the concentration of neutrophils, eosinophils, basophils, dendritic cells (DCs), and natural killer cells (NK cells) (Fig. 1A–C,H,I) did not differ between healthy controls and patients, nor it changes during therapy treatment.

**Figure 2:**
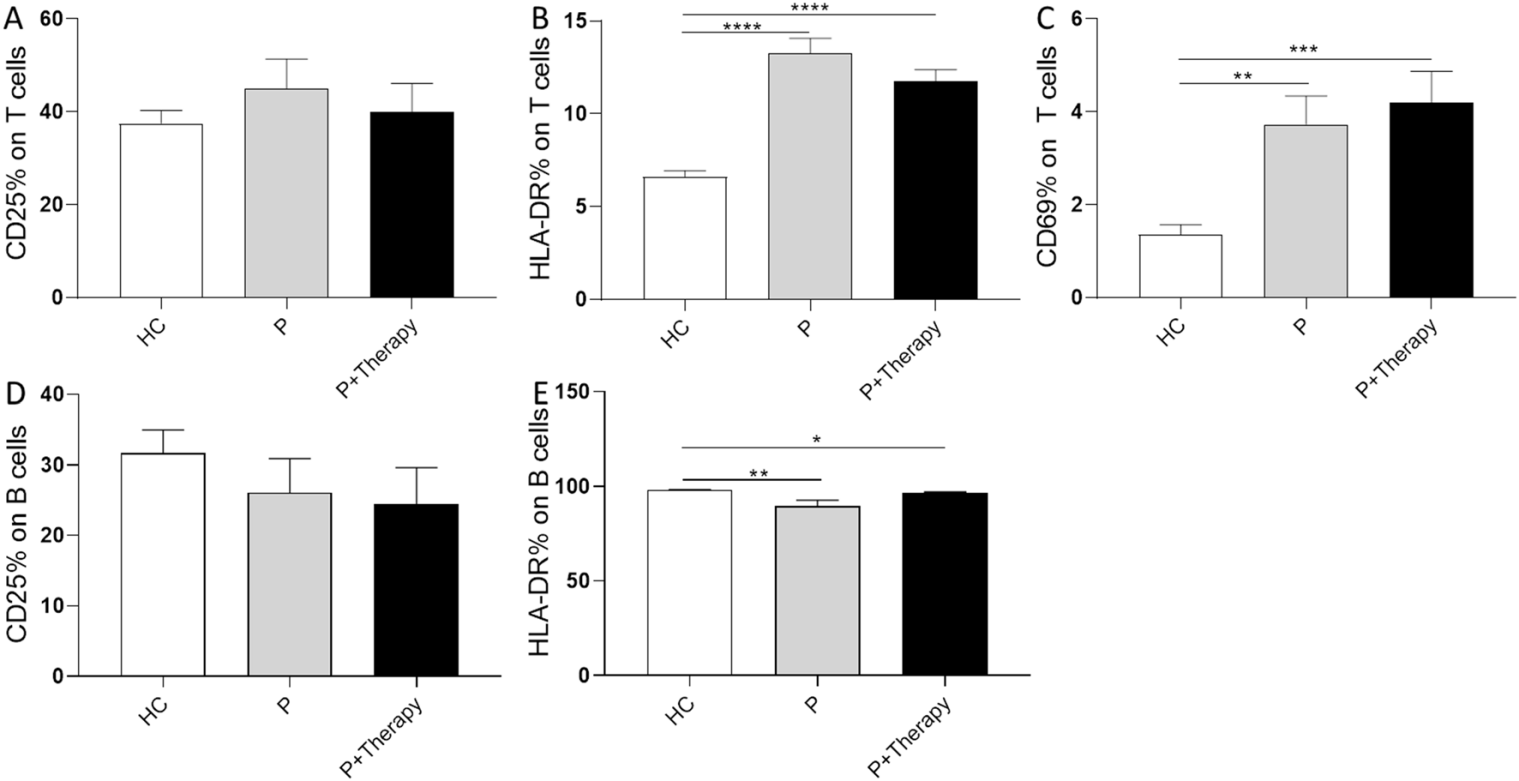
Analysis of activation markers on subsets of peripheral blood cells. The activation markers CD25, CD69 and HLA-DR were analysed for their expression on peripheral blood cell subtypes T cells and B cells in healthy controls (HC) and patient blood before their first (P) and after second therapy treatment (P + Therapy). (**A**) CD25 on T cells is slightly downregulated in OA patients after second treatment. (**B**) The percentage of HLA-DR on B cells is slightly upregulated in OA patients compared with healthy controls after treatment. (**C**) CD69 on T cells shows an increase in OA patients after therapy treatment. Overall, this activation marker is upregulated in OA patients. (**D**) CD25 on B cells shows a slight decrease in OA patients after treatment. (**E**) HLA-DR on B cells is significantly downregulated in OA patients but increases after therapy treatment. Results are given as mean ± SEM; T-test, *p < 0.5; **p < 0.01; ***p < 0.001; ****p < 0.0001.

A significant decrease in the concentration of CD4^+^ T cells after the second therapy treatment (595.2 ± 77.8 × 10^3^ cells/ml) compared with healthy controls (973.9 ± 79.2 × 10^3^ cells/ml) (Fig. 2E) can be seen. Like CD4^+^ T cells, T_reg_ cells showed a significant decrease when comparing the treatment group (43.2 ± 5.9 × 10^3^ cells/ml) with the subject group (59.3 ± 4.1 × 10^3^ cells/ml) (Fig. 1F). B cells (Fig. 1G) were significantly decreased in both OA groups compared to the control subject group (Ctrl: 258.6 ± 27.4, OA: 107.8 ± 29.6, Therapy: 107.0 ± 31.0 × 10^3^ cells/ml). Only one cell type showed a significant difference between the OA group and the therapy group. Monocytes (Fig. 1D) were increased with a significant difference between OA group (450.4 ± 102.3 × 10^3^ cells/ml) and the therapy-treatment group (571.1 ± 77.8 × 10^3^ cells/ml).

Furthermore, the expression of the activation markers CD25, CD69 and HLA-DR on T cells, B cells, NK-cells, and monocytes of patients and healthy controls (Fig. 2) was investigated. In summary, no significant modulating effects of this therapy on the expression of activation markers on peripheral blood cells was seen. However, differences in the activation state of immune cells between healthy controls and patients were detected. Specifically, the percentage of CD69 on T cells was significantly increased in OA patients compared with healthy controls (Fig. 2C), as well as the expression of HLA-DR on T cells (Fig. 2B). Further HLA-DR was significantly downregulated on B cells from OA patients compared with healthy controls (Fig. 2E). Additionally, the percentage of CD25 on B and T cells was slightly, although not significantly, decreased in OA patients after treatment compared with before (Fig. 2A,D). Analysis of monocyte and NK cell activation markers showed no significant modulations (data not shown).

**Figure 3:**
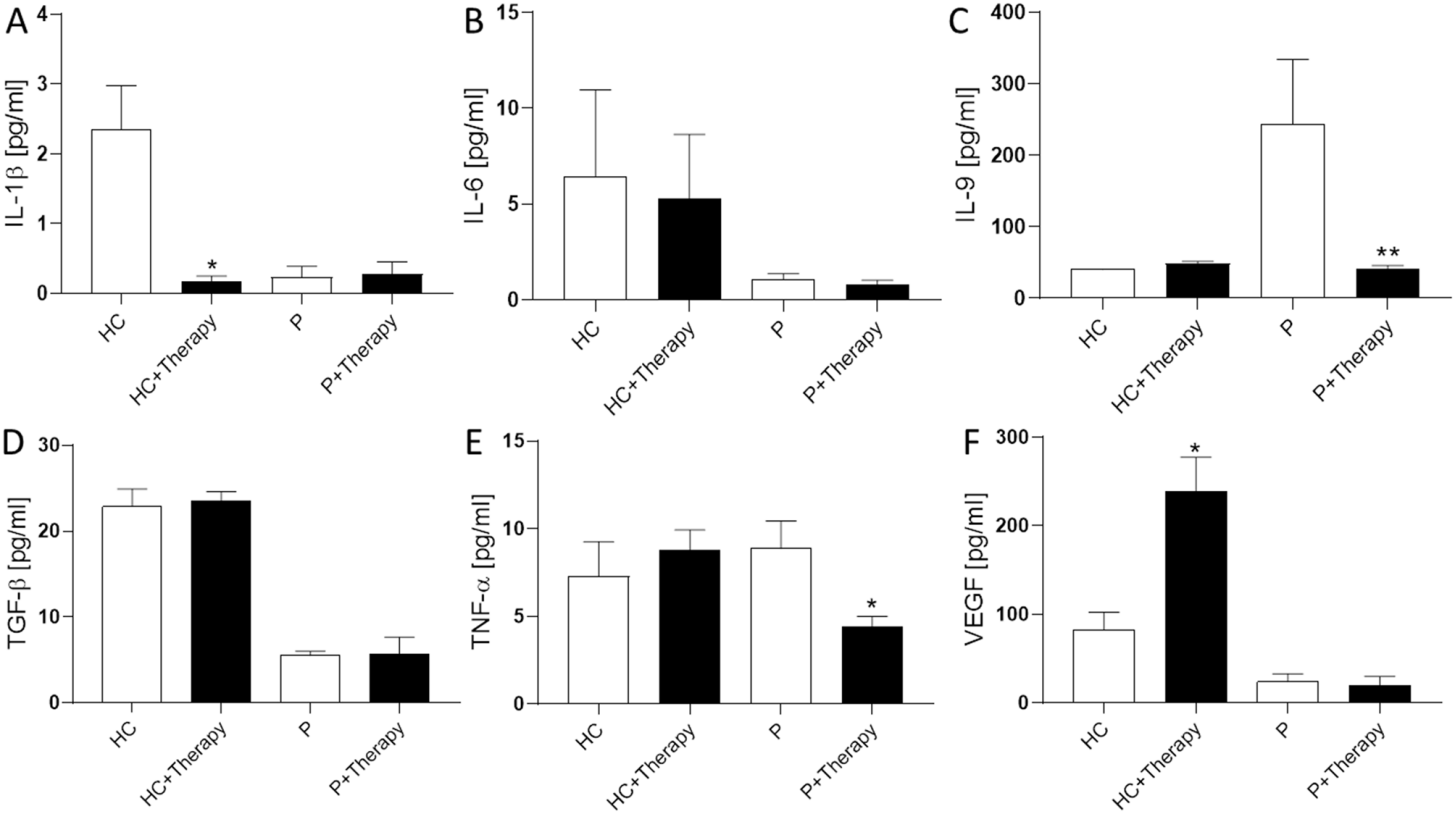
Cytokine concentrations in serum before and after Goldic therapy. (**A**) IL-1β concentration decreases in healthy controls serum (HC) after incubation with gold-particles (HC + Therapy), but showed no effect in patients (P + Therapy). (**B**) IL-6 and TGF-β (**D**) did not change because of therapy treatment in either healthy controls or patients. (**C**) IL-9 is upregulated in patients (P) but could be downregulated to normal levels after treatment. (**E**) TNF-α is significantly upregulated in healthy controls, but significantly downregulated in patients after treatment. (**F**) VEGF is significantly upregulated in healthy controls after treatment, but showed no change in patients. Results are given as mean ± SEM; T-test, *p < 0.5; **p < 0.01.

In addition to peripheral blood cell composition, it was investigated whether the concentration of cytokines in the serum of healthy controls and patients differs after therapy treatment. For this purpose, incubated whole blood samples with gold and decanted the serum were included for analysis. The concentration of IL-1β in healthy controls decreased significantly after therapy treatment from 2.5 ± 0.6 in HC to 0.1 ± 0.08 pg/ml in HC + Therapy (Fig. 3A). In the serum of patients, the concentration of IL-1β was lower and remained stable. The amount of IL-6 (Fig. 3B) did not change significantly with therapy treatment in the healthy controls (HC: 57.7 ± 8.7, HC + Therapy: 74.8 ± 11.1 pg/ml). Compared with the healthy controls, the IL-6 concentration of the patients was lower and did also not change by therapy treatment. IL-9 (Fig. 3C) showed no modulation by therapy in the healthy controls (HC: 40.1 ± 6.4, HC + Therapy: 48.2 ± 3.3 pg/ml). But in patients, IL-9 was significantly reduced following therapy treatment. Significant modulation of TGF-β concentration (Fig. 3D) is also not found in either healthy controls (HC: 22.9 ± 2.0, HC + Therapy: 23.5 ± 1.0 pg/ml) nor patients (P 5.5 ± 0.4, P + Therapy 5.6 ± 1.9 pg/ml). Both TNF-α (Fig. 3E) and VEGF (Fig. 3F) concentrations showed a significant increase after therapy in the sample serum. TNF-α concentration increases from 5.31 ± 0.30 pg/ml in HC to 7.6 ± 0.5 pg/ml in therapy serum (HC + Therapy). But in patients a significant reduction of TNF-α from 8.9 ± 1.5 pg/ml in P to 4.4 ± 0.5 pg/ml in P + Therapy was detected. For VEGF, an increase from 82.9 ± 19.4 in HC to 238.5 ± 38.8 pg/ml in HC + Therapy, as well as a reduction in patient serum from 40.9 ± 18.6 in P to 25.4 ± 10.4 pg/ml in P + Therapy was seen.

## Discussion

The main finding of this study is the downregulation of inflammation related cytokines in OA patients after treatment with an autologous gold-based serum therapy (Fig. 3). Overall cytokine levels did not show high modulations because of therapy-treatment. Significant downregulations were seen in IL-9 and TNF-α (Fig. 3C,E). IL-9 secretion, which is closely related to Th_9_ cells associated with pro-inflammatory and degenerative processes in diseases such as rheumatoid arthritis, is significantly upregulated in the serum of OA patients^9^. Here, a significant reduction in serum IL-9 levels in OA patients after two treatments with autologous serum therapy (Fig. 3C) are detected. Moreover, the concentration of TNF-α, which is associated with cartilage degeneration and pro-inflammatory processes in OA and a target of various pharmaceutical agents^10–12^, is significantly downregulated in therapy-treated patients (Fig. 3E). These results show promising modulations of patient’s serum cytokine levels and represent a new approach to address the degenerative and proinflammatory processes involved in the progression of OA.

The peripheral blood cell composition did not show any modulation in neutrophils (Fig. 1), basophils, eosinophils, dendritic cells or natural killer cells after second treatment with the gold-based ACS. For neutrophil granulocytes and eosinophils an increase would been expected, as these cells are involved in cartilage degradation^13^ and are activated in the setting of arthritis, but was not seen at this state. Additionally these cells are thought to have an influence on synovial macrophages by shifting them from an anti-inflammatory to pro-inflammatory state to have an intrinsic influence on the course of arthritis and are often reported to be upregulated in patients^14^. This can be related to the type or stage of arthritis and to the fact that OA patents have lower amounts of eosinophils than patients suffering from other forms of arthritis. The role of this peripheral cell subtype needs to be reconsidered, as in an experimental approach of rheumatoid arthritis eosinophils appear to have a resolving function^15^.

In monocytes (Fig. 1D) a significant upregulation after treatment with the autologous therapy was seen. This cell type, which is associated with cartilage degeneration and inflammation, is critically involved in the pathology of OA and a decrease is considered to be beneficial^16^. However, there are anti-inflammatory cell subtypes of monocytes that must be scrutinized. These cells have the ability to produce anti-inflammatory mediators and to suppress the activation of T cell subtypes^17^. In addition, monocyte classification experiments are needed to understand how this increase in monocytes in peripheral blood is caused and how it affects T cells. For CD4^+^ T cells (Fig. 1E) an overall decrease in OA patients can be seen. Higher levels of CD4^+^ T cells normally are detectable in peripheral blood, which is associated with the pathology of arthritis^18,19^. The stage of OA can be an important factor for the analysis of the amount of CD4^+^ T cells, because late-stage OA has a lower amount of T cells in the peripheral blood observation was seen in T_reg_-cells (Fig. 1F) and B cells (Fig. 1G). Previous studies on peripheral blood composition stated that B cell levels are lower in OA patients than in healthy controls^20^.

Significant regulations of activation markers were most visible in the T cell compartment. In this study, CD69, an early activation marker, and HLA-DR, which is involved in antigen presentation, were upregulated on the T cells of OA patients compared with healthy controls^21–23^. Accordingly, increased numbers of activated T cells are also found in the synovium and infrapatellar fat pad of patients with knee OA^22^. Even though lower numbers of T cells were found in OA patients, these cells show a more active phenotype as they play a central role in OA. A slight but significant downregulation of HLA-DR is found on B cells from patients compared to healthy controls. This downregulation of HLA-DR could indicate an impaired B cell immune response. In addition, a light downregulation of CD25 on T cells and B cells after therapy treatment in patients was detectable. This modulation suggests that the therapy can modulate not only cell numbers but also the activation status of cells of the adaptive immune system.

The primary results presented of this gold-based ACS treatment for OA are based on peripheral blood and cytokine data. These data just give a first insight of the therapy background and must be validated by larger cohorts of patients and healthy controls. In addition, new experiments need to incorporate synovial fluid analysis to investigate how cells migrate during disease and how the ACS contributes in this process. Furthermore, time points need to be increased and analysis of the blood cells and cytokine levels measured after further treatments with the ACS. In addition, surveys of the pain felt by patients and medical examinations of the overall health of the affected joints need be included to see the benefit of this therapy for OA patients.

Most cell types analysed showed no significant modulation after the second therapy treatment in patients, but a significant decrease in T and B cells is seen in OA patients. The pro-inflammatory cytokines IL-9 and TNF-α were significantly downregulated in patient serum after second therapy treatment. Further experiments and larger cohorts of patients and healthy controls are needed to investigate the effects of gold-based ACS.

## Methods

### Blood and serum samples

#### Healthy controls

**Figure 4:**
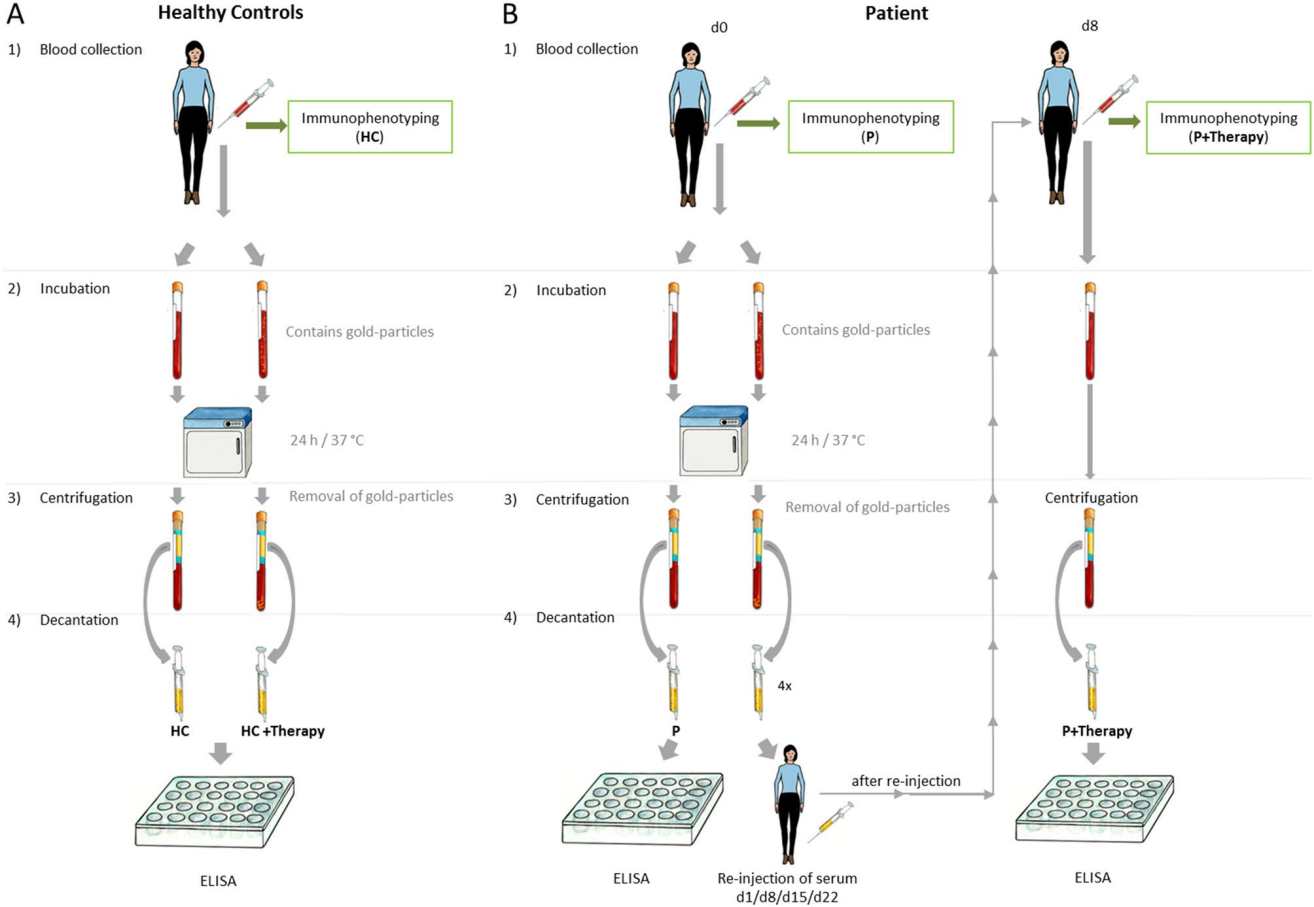
Acquisition of serum and whole blood samples from healthy controls and patients. (**A**) Blood samples were collected from healthy controls and patients (**B**). One whole blood sample was sent directly for immunophenotyping. Additional blood samples were placed into tubes containing gold-microparticles and incubated at 37 °C for 24 h. After incubation, samples were centrifuged to remove microparticles and blood cells, and serum was decanted. The decanted serum was used for further analysis. In addition, the serum was re-injected into the patients for therapy treatment. *After the second treatment with autologous serum (before they received the third injection) another sample was generated for analysis. CC By Jörg Pekarsky.

Whole blood samples were collected from healthy controls by medical personnel after their consent (Fig. 4). The healthy controls (HC) had no known form of osteoarthritis or other inflammatory diseases. A total of 18 ml of blood was collected for immunophenotyping (Fig. 4A) and treatment with gold particles. For the gold treatment, the blood was directly placed into a syringe containing gold-microparticles (1 µm radius) and was incubated at 37 °C for 24 h (Fig. 4A). A gold-particle concentration of 10^3^–10^4^ (gold micro carriers, BioRad Laboratories, Cat#165–2264) on average per 10 ml of blood was chosen as the working concentration. After incubation, blood samples were centrifuged, serum was decanted and used for further analysis. Blood was collected from ten healthy controls (2 male; 8 female; 26–65 years). No healthy control came into direct contact with the particles (re-injection of serum).

#### Patients

Whole blood samples were taken from patients by medical personnel after their consent. Patients declared that they were not suffering from other inflammatory diseases or taking anti-inflammatory drugs. A total of 18 ml of blood was drawn from the patients for immunophenotyping (Fig. 4B) and serum analysis before their first therapy treatment (P) as a control and after the second injection of therapy (P + Therapy). Blood from six (5 male, 1 female from 53 to 82 years) OA patients was collected. Whole blood samples were analysed by immunophenotyping 24 h after collection. The therapeutic serum/blood was placed in 10 ml collection containing gold-microparticles (1 µm radius) and incubated at 24 h at 37 °C (Fig. 4B). A gold-particle concentration averaging 10^3^–10^4^ (gold micro carriers, BioRad Laboratories, Cat#165-2264) per 10 ml of blood was chosen as the working concentration. The protocol and therapy are protected by patent EP2590659B1. After incubation, blood samples were centrifuged and serum was decanted and used for further analysis (Fig. 4B [4]). The ethics committee of the University Erlangen-Nuremberg (60_19 B) approved this study which was made under compliance of the Helsinki Declaration. The results were presented as a pool of all individually tested sera.

### Immunophenotyping

100 µl whole blood samples were analysed by multi-colour flow cytometry for 20 different immune cell subtypes and three common activation markers to determine a general immune status. Staining was performed according to previously published protocols for modular immunophenotyping (IPT)^24,25^. IPT was performed approximately 24 h after whole blood collection. The immune status of patients was compared with healthy controls. Data acquisition was performed on a Gallios Flow Cytometer (Beckman Coulter) in the standard filter configuration. Kaluza^®^ Flow Analysis Software (Beckman Coulter) was used for data analysis.

### Enzyme-linked immunosorbent assay (ELISA)

ELISAs were purchased from R&D Systems as a human DuoSet sandwich ELISA and performed according to the manufacture’s protocol (IL-1β DY-201, IL-6 DY-205, IL-9 DY-209, TNF-α DY210, TGF-β DY240, VEGF DY293B). Plates were coated with the capture antibody the evening before. The next morning, the plate was washed three times with PBS-T and blocked with 1% BSA-PBS for 1 h at room temperature. After blocking and washing, samples and standard were added for 2 h at room temperature. After incubation, the plate was washed three times and incubated with the detection antibody for 2 h at room temperature. After another washing step, the plate was incubated with streptavidin/HRP for 20 min in the dark. The plate was washed and TMB solution was added for colorimetric analysis. The reaction was stopped and the optical density was measured at 490 nm. Samples were normalized to 10 µg/µl of total protein for better comparability.

### Statistics

The results were analysed by t-test. Results are given as mean ± SEM and p-values below 0.05 are considered significant. Calculation and visualization was performed with GraphPad Prism 6 (GraphPad Prism software; GraphPad Software, Inc., San Diego, CA, USA).

### Informed consent

Informed consent was obtained from all healthy controls as well as from patients before collecting blood samples.
